# Difficulty With Binary Voting Among FDA Oncology Advisory Committee Members

**DOI:** 10.1001/jamanetworkopen.2025.22759

**Published:** 2025-07-23

**Authors:** Kaatje S. Greenberg, Joshua Hopen, Muhammad Draw, Matthew S. McCoy, Genevieve P. Kanter, Holly Fernandez Lynch

**Affiliations:** 1 Carey Law School, University of Pennsylvania, Philadelphia; 2 Department of Medical Ethics and Health Policy, Perelman School of Medicine, University of Pennsylvania, Philadelphia; 3 Leonard Davis Institute of Health Economics, University of Pennsylvania, Philadelphia; 4 Department of Health Policy and Management, Sol Price School of Public Policy, University of Southern California, Los Angeles; 5 Leonard D. Schaeffer Center for Health Policy and Economics, University of Southern California, Los Angeles

## Abstract

This qualitative study investigates the frequency of reports of difficulty in voting for drug approval by members of the Food and Drug Administration Oncologic Drugs Advisory Committee.

## Introduction

The US Food and Drug Administration (FDA) convenes advisory committees to inform complex drug approval decisions, with substantial alignment between committee votes and FDA actions.^1^ These meetings typically culminate with members voting on FDA’s questions, followed by members explaining their positions. There has been debate about the utility of committee votes, which may not capture substantive discussion or member uncertainty.^2^ To assess this potential limitation, we examined how frequently members of the Oncologic Drugs Advisory Committee (ODAC), one of FDA’s most frequently convened advisory committees, described binary voting as difficult and their reasons.

## Methods

In accordance with the Common Rule, institutional review board approval and informed consent were not required for this qualitative study because advisory meetings are public. We followed the SRQR reporting guideline.

We retrieved ODAC transcripts from January 2008 to October 2022 from FDA’s website, analyzing type of voting question (eg, whether the drug “should be approved” or “has a favorable risk-benefit profile”), whether members expressed difficulty in explanatory statements, and, if so, their articulated rationale for difficulty (eMethods and eAppendix in Supplement 1). We defined voting difficulty as any noted struggle deciding how to proceed on a binary vote (eg, explicit acknowledgment of difficulty or expressions of reluctance, hesitance, uneasiness, or internal conflict), excluding mere acknowledgment of complexity. Recognizing that difficulty may present along a spectrum, we erred on the side of inclusion to capture all potentially difficult decisions. We double coded all voting statements (K.S.G. and J.H.), with a third reviewer (H.F.L.) resolving infrequent disagreement, and tabulated frequency of difficult votes and rationales. We used generalized estimating equations, accounting for within-person correlations, to evaluate whether question type, member type, or vote direction (ie, in favor of or against the sponsor) were associated with difficulty.

## Results

During the sample period, ODAC convened 71 voting sessions, in which 230 scientific members, 45 patient representatives, and 12 consumer representatives participated in at least 1 vote on 83 questions. Of 1021 member votes, 990 votes (97.0%) included explanatory statements. Fewer than 1 in 10 statements (92 statements [9.3%]) indicated a difficult voting decision, without any discernable pattern by year. The most frequent rationales for difficulty were unconvincing post hoc analysis (28 statements [30.4%]) and unmet need (23 statements [25.0%]) (Table 1).

**Table 1.** Rationales Given for Vote Difficulty

Rationale^a^	Exemplary quotation	Statements indicating difficulty by vote direction, No. (%)			
		Overall (n = 92)	In favor of approval (n = 35)	Against approval (n = 46)	Abstained (n = 11)
Issues with data					
Unconvincing post hoc analysis	“I had some difficulty, as others have, in coming to a decision….[M]y main concerns were the doubts that were raised about the design and interpretation of the results.”	28 (30.4)	7 (20.0)	19 (41.3)	2 (18.2)
Poor study design	“I didn’t feel like this was a real solid yes. I was concerned about methodological problems.”	14 (15.2)	2 (5.7)	9 (19.6)	3 (27.3)
Not enough data	“I was on the fence. Again, I would have preferred a non-binary vote, maybe something fluid on the Likert, say from 1 to 10 but that would be more difficult for the FDA to analyze….I want to see additional data.”	13 (14.1)	4 (11.4)	9 (19.6)	0
Need postmarket data	“I voted yes, also very reluctantly….I would urge the sponsor in postmarketing evaluations to more fully understand both the hypothyroidism…[and] the VTE.”	9 (9.8)	6 (17.1)	3 (6.5)	0
Efficacy for subpopulation	“I’m really on the fence because I could see how this would be beneficial to some patients but not the ones in this study as was demonstrated here.”	8 (8.7)	2 (5.7)	5 (10.9)	1 (9.1)
Real-world implications	“It was 51-49 in my head, and ultimately I was concerned a lot about some real-world problems that we are now going to face.”	3 (3.3)	1 (2.9)	2 (4.3)	0
Drug meets an unmet need	“[I]t was a difficult decision, and it really came down to the fact that where we’re at right now with many patients, they need something[.]”	23 (25.0)	15 (42.9)	8 (17.4)	0
Substantial risk involved	“I was very conflicted on this vote....I am most concerned about a possible signal that this drug may cause…a serious toxicity and could actually lead to early death[.]”	12 (13.0)	5 (14.3)	7 (15.2)	0
Issues with question phrasing	“I hate this process of voting on this question because I believe in the drug. I think it works. I think it benefits patients. I think it should be approved. But the language of the question is do I believe the benefits outweigh the risks?”	7 (7.6)	4 (11.4)	2 (4.3)	1 (9.1)
Deference to other committee members	“I defer to the lymphoma people who know more about this disease than I do.”	3 (3.3)	1 (2.9)	0	2 (18.2)
No identifiable rationale given	“I abstained. I think every situation – every situation is unique and I think that’s what we have…the Phase II meetings for.”	1 (1.1)	0	0	1 (9.1)
Other (eg, feasibility of confirmatory trials or small clinical trial size due to rare disease)	“I’m still concerned, still uneasy with the fact that the patient population in which this drug was tested is very small in the U.S. And I was really hoping to hear from the audience patient experiences with this particular application of this drug….So yes but with hesitation.”	9 (9.8)	5 (14.3)	3 (6.5)	1 (9.1)

^a^ Committee members could identify more than 1 rationale for their decision.

There were significant differences in how often distinct question types were deemed difficult (Table 2). Questions about risk-benefit profile, whether a randomized trial should be required for approval, or whether an approved indication should be maintained or withdrawn were more likely to elicit voting difficulty than direct questions about whether a drug should be approved.

**Table 2.** Association of Question Type, Vote Direction, and Member Type With Self-Reported Voting Difficulty

Factor	Observations, No. (%)	Marginal probability of voting difficulty vs reference category (95% CI), percentage points^a^	*P* value
Type of question			
Observations, No.	982	NA	NA
Does the drug have a favorable risk-benefit profile?	556 (56.6)	4.89 (6.30 to 9.14)	.02
Is a randomized controlled trial needed?	79 (8.0)	10.67 (3.41 to 17.93)	.004
Should the indication be withdrawn/maintained?	65 (6.7)	14.74 (4.14 to 25.34)	.006
Do the data support efficacy?	103 (10.5)	−0.01 (−5.81 to 4.76)	.85
Should further evaluations be required?	46 (4.7)	3.64 (−5.69 to 12.97)	.45
Probability of voting difficulty of: Should the drug be approved (reference category) (95% CI), %	133 (13.5)	4.41 (0.8 to 8.0)	NA
Direction of vote			
Observations, No.	979	NA	NA
Favor sponsor	489 (49.9)	−2.91 (−6.59 to 0.77)	.12
Probability of voting difficulty of: Do not favor sponsor (reference category) (95% CI), %	490 (49.9)	9.57 (6.31 to 12.83)	NA
Member type^b^			
Observations, No.	990	NA	NA
Chairperson	78 (7.9)	3.76 (−3.48 to 11.00)	.31
Consumer representative	78 (7.9)	6.57 (−3.52 to 16.68)	.20
Patient representative	76 (7.7)	−2.40 (−8.62 to 3.82)	.45
Probability of voting difficulty of: Scientific member (reference category) (95% CI), %	758 (76.6)	8.63 (6.31 to 10.94)	NA

Abbreviation: NA, not applicable.

^a^ Votes were deemed *not difficult* if the member’s voting statement did not indicate voting difficulty. The marginal probability of a vote being difficult was estimated using generalized estimating equations with a logit link, specifying an exchangeable working correlation structure and robust standard errors to account for within-person correlations across questions and meetings. Type of question analysis excluded 8 observations associated with the other category because there was no variation in vote difficulty for this question type. Direction of vote analysis excluded 11 abstention votes, which were by definition coded as difficult.

^b^ Scientific members include standing and temporary voting members and exclude chairpersons.

Voting difficulty was not associated with probability of voting for or against the sponsor (Table 2). Additionally, there was no significant difference in probability of voting difficulty by member type (Table 2).

## Discussion

High rates of difficulty could indicate that binary voting fails to accurately capture advisory committee member perspectives. However, this qualitative study found that relatively few ODAC votes were difficult. As the FDA contemplates potential reform,^3,4^ these results suggest that voting difficulty is not an independent reason to alter binary voting. Numerical voting, along a confidence scale or as an indication of strength of support, could offer more nuance but may be harder to interpret.^5^

Voting difficulty varied significantly by question type. One concern is that questions that appear closely linked (eg, about approval and risk-benefit profile^6^) elicited different levels of difficulty. The FDA should reconsider question types that frequently elicit difficulty, with attention to members’ generalizable concerns.

Although our analysis was limited to a single advisory committee, neither question types nor voting rationales observed for ODAC appeared unique to the oncology setting. Designating votes as difficult required interpretation but in most cases was straightforward. Our analysis excluded other meeting discussion beyond voting statements where difficulty may have been expressed. However, nearly all member votes included explanatory statements, with a meaningful number indicating difficulty, suggesting that failure to do so reasonably indicated confidence. Overall, these findings suggest that binary voting can accurately capture advisory committee perspectives in most cases.

## Notes

Supplement 1.
eMethods.
**eAppendix.** Codebook Used to Analyze Voting Statements

Supplement 2.
Data Sharing Statement
