# Supplementary material for: Difficulty With Binary Voting Among FDA Oncology Advisory Committee Members
Source: JAMA Netw Open. 2025 Jul 23;8(7):e2522759. doi: 10.1001/jamanetworkopen.2025.22759 (PMC12287828; doi:10.1001/jamanetworkopen.2025.22759)
Supplement: Supplement 1. — eMethods. eAppendix. Codebook Used to Analyze Voting Statements [file jamanetwopen-e2522759-s001.pdf]

Supplemental Online Content

Greenberg KS, Hopen J, Draw M, McCoy MS, Kanter GP, Lynch HF. Difficulty with binary voting among FDA oncology advisory committee members. *JAMA Netw Open*. 2025;8(7):e2522759. doi:10.1001/jamanetworkopen.2025.22759

eMethods.
eAppendix. Codebook Used to Analyze Voting Statements

This supplemental material has been provided by the authors to give readers additional information about their work.

© 2025 Greenberg KS et al. *JAMA Network Open*.

eMethods

This study employs a blended approach, integrating qualitative and quantitative methods. We sourced our primary data from meeting transcripts, video recordings, and supplementary materials corresponding to voting sessions of the US Food and Drug Administration (FDA) Oncologic Drug Advisory Committee (ODAC) between January 2008 and October 2022. This time period includes all transcripts available at the time of data collection. All materials are readily accessible to the public on the FDA website (FDA.gov).

We manually extracted all voting questions, votes/abstentions, and voting statements from each ODAC meeting transcript. Additional data, including the committee member's name and member type (e.g., standing member or patient representative), were also recorded. Once data collection was complete, we assessed each voting question to determine whether a "yes" vote indicated support for or opposition to the sponsor's application, allowing comparisons between question types.

Qualitative Analysis

We generated a preliminary codebook by examining a sample of approximately 10% of the 990 voting statements in the dataset. (Appendix A2). Each voting statement was assigned a confidence code – either "difficult decision" or "confident decision" (i.e., decision that was not difficult). A statement was coded as a difficult decision if the voting member expressed a struggle in deciding whether to vote "yes" or "no" in answer to the voting question, beyond acknowledging that the issue before the committee was complex; struggle was typically evidenced through language explicitly referencing difficulty in deciding whether to vote "yes" or "no" or expressing feelings of hesitance, reluctance, unease, or internal conflict about their vote. Abstentions were coded as difficult decisions. A statement was coded as a confident decision if there was no indication that the member struggled with binary voting. To ensure comprehensive inclusion of all voting statements that could reasonably be described a difficult, edge cases were coded as difficult. We also generated codes to capture the nature of the question posed to voters (e.g., asking whether the drug should be approved; whether the voter believed the drug's risk/benefit profile was favorable; or whether further evaluations of the drug were needed prior to approval).

Once the question type codebook was finalized, two authors applied the codebook to double code another ~20% of the dataset. Codes were entered into separate columns in an Excel workbook, with each coder's entries hidden from the other. The coders met to compare results and to refine the codebook. We then continued to double code the remainder of the dataset. Double coding was used for all qualitative data analysis to promote consistency. Any discrepancies were resolved through discussion between the coders and, when needed, discussion with a third member of the research team who was not engaged in coding, minimizing the risk of interpretation bias.

After coding the confidence and question type of each statement, we generated a second preliminary codebook to capture rationale themes within the subset of data coded as "difficult decisions" (e.g., the voter's struggle coming to a decision turned on the substantial risks of the drug; the likelihood that the drug would meet unmet patient need; or issues with the study design or post-hoc analysis of study data). Rationale themes emerged inductively from the data; we did

not approach the rationales with any pre-specified codes in mind. We used the same method as described above to apply this second codebook to all difficult decisions.

Quantitative Analysis

We tabulated the number of explanatory statements by self-reported vote difficulty. We also tabulated the distribution of the rationales that members provided for why a decision was difficult, overall and by whether the vote was in favor of the sponsor or against. The small sample size of difficult decisions (n=92) precluded statistical analysis for these latter tabulations.

To quantify the association between vote difficulty and question type, voting in favor of the sponsor, and member type, we used Generalized Estimating Equations (GEE) to account for the correlation structure. In our sample, members voted for multiple questions within a meeting, and some (but not all) members voted in multiple meetings. We used GEE with a logit link function, specifying an exchangeable working correlation structure and using robust standard errors.

© 2025 Greenberg KS et al. *JAMA Network Open*.

eAppendix. Codebook Used to Analyze Voting Statements

Decision Difficulty; Question Type; Decision Rationale
**Decision Difficulty** *(only one category may apply)*<br><br>Difficult Decision: Voting member expresses a struggle in deciding whether to vote "yes" or "no" in answer to the voting question, beyond acknowledging that the issue is complex; abstentions are an indicator of difficulty.<br><br>Confident Decision: Voting member does not express a struggle in their decision, even if they acknowledge that the issue is complex.
**Question Type** *(only one category may apply)*<br><br>Does the drug have a favorable risk-benefit profile? Question asks voting members whether the drug has a favorable risk-benefit profile or whether the drug's benefits outweigh its risks.<br><br>Do the data support efficacy? Question asks voting members whether the available data support the drug's efficacy, results are reasonably likely to predict clinical benefit, the response is clinically meaningful, or results confirm benefit for the population(s) or indication(s) for which approval is being sought.<br><br>Should the drug be approved? Question asks voting members whether the drug should be approved, including questions about accelerated approval, or licensed, including questions about biosimilars.<br><br>Is a randomized controlled trial needed? Question asks voting members explicitly whether a randomized trial should be conducted or completed prior to approval.<br><br>Should further evaluations be required? Question asks voting members whether further evaluations (other than specific mention of a randomized trial) should be required prior to a regulatory decision or after approval.<br><br>Should the indication be withdrawn/maintained? Question asks voting members whether an existing approved indication(s) should be withdrawn from the drug's labeling or maintained, including based on existing evidence or pending additional study.<br><br>Other: Question does not fall into any of the above categories (the sole question in this category asked whether an additional diagnostic test should be required prior to approval).
**Difficult Decision Rationale** *(more than one category may apply)*<br><br>Substantial risk involved: Voting member is troubled by the drug's considerable risks.<br><br>Drug meets an unmet need: Voting member believes that, if approved, the drug will provide a treatment option for patients who have few or no existing options.<br><br>Poor study design: Voting member identifies flaws in the study design of completed trials.

© 2025 Greenberg KS et al. *JAMA Network Open*.

Unconvincing post hoc analysis: Voting member identifies insufficiencies in the sponsor's analysis of completed trials (e.g., premature or conclusory data) such that the voting member cannot infer meaningful results.

Not enough data: The completed studies and trials, though not themselves flawed, are insufficient to allow the voting member to draw conclusive inferences.

Efficacy for subpopulation: Voting member suggests that the drug may be beneficial for a subpopulation of the proposed indication(s) or for another indication entirely, but not for the sponsor's proposed indication(s).

Real world implications: Voting member expresses concern or doubt about the generalizability of the trial results or otherwise expresses concern about the drug's use in a broader patient population.

Need post-market data: Voting member believes further trials are needed after approval to fill evidentiary gaps or otherwise references confirmatory studies.

Issues with question phrasing: Voting member believes the voting question is unclear, confusing, misleading, or otherwise challenging to answer.

Deference to other committee members: Voting member believes other committee members are better suited to answer the voting question and chooses to rely on their statements.

No identifiable rationale given: Voting member expresses difficulty in deciding how to vote, but voting statement does not express a clear rationale for that difficulty.

© 2025 Greenberg KS et al. *JAMA Network Open*.
