# Supplementary material for: Difficulty With Binary Voting Among FDA Oncology Advisory Committee Members
Source: JAMA Netw Open. 2025 Jul 23;8(7):e2522759. doi: 10.1001/jamanetworkopen.2025.22759 (PMC12287828; doi:10.1001/jamanetworkopen.2025.22759)
Supplement: Supplement 2. — Data Sharing Statement [file jamanetwopen-e2522759-s002.pdf]

Data Sharing Statement

Greenberg. Difficulty With Binary Voting Among FDA Oncology Advisory Committee Members. *JAMA Netw Open*. Published online July 23, 2025. doi:10.1001/jamanetworkopen.2025.22759

Data
Data available: Yes
Data types: Data (not involving human participants)
How to access data: Data will be provided upon request to lynchhf@pennmedicine.upenn.edu.
When available: With publication

Supporting Documents
Document types: None

Additional Information
Who can access the data: Researchers whose proposed use of the data has been approved.
Types of analyses: Any legitimate academic purpose.
Mechanisms of data availability: Data will be provided via email without investigator support.
